# Ketamine Clinical Use on the Pediatric Critically Ill Infant: A Global Bibliometric and Critical Review of Literature

**DOI:** 10.3390/jcm12144643

**Published:** 2023-07-12

**Authors:** Mary Lucy Ferraz Maia, Lucas Villar Pedrosa Silva Pantoja, Brenda Costa Da Conceição, Kissila Márvia Machado-Ferraro, Jackeline Kerlice Mata Gonçalves, Paulo Monteiro Dos Santos-Filho, Rafael Rodrigues Lima, Enéas Andrade Fontes-Junior, Cristiane Socorro Ferraz Maia

**Affiliations:** 1 Laboratory of Pharmacology of Inflammation and Behavior, Faculty of Pharmacy, Institute of Health Science, Federal University of Pará, Belém 66075-900, Pará, Brazil; mary.maia@icb.ufpa.br (M.L.F.M.); kissila.machado@ics.ufpa.br (K.M.M.-F.); jackelinekerlice@gmail.com (J.K.M.G.); efontes@ufpa.br (E.A.F.-J.); 2 Laboratory of Functional and Structural Biology, Biological Science Institute, Federal University of Pará, Belém 66075-110, Pará, Brazil; rafalima@ufpa.br

**Keywords:** ketamine, sedation, bibliometric study, sedoanalgesia, pediatric intensive care units, pediatric patient

## Abstract

The developing central nervous system is vulnerable to several stimuli, especially psychotropic drugs. Sedation procedures during the developmental period are frequent in pediatric intensive care units (PICUs), in which the use of the sedative agent is still a challenge for the PICU team. Ketamine has been indicated for sedation in critically ill children with hemodynamic and ventilatory instabilities, but the possible neurobehavioral consequences related to this use are still uncertain. Here, we performed a bibliometric analysis with conventional metrics and a critical review of clinical findings to reveal a gap in the literature that deserves further investigation. We revealed that only 56 articles corresponded to the inclusion criteria of the study. The United States of America emerges as the main country within the scope of this review. In addition, professional clinical societies play a key role in the publications of scientific clinical findings through the specialist journals, which encourages the sharing of research work. The co-occurrence of keywords evidenced that the terms “sedation”, “ketamine”, and “pediatric” were the most frequent. Case series and review articles were the most prevalent study design. In the critical evaluation, the scarce studies highlight the need of use and post-use monitoring, which reinforces the importance of additional robust clinical studies to characterize the possible adverse effects resulting from ketamine anesthetic protocol in critically ill children.

## 1. Introduction

In pediatric critical patients, intensive care units usually use sedation associated with analgesia to change the level of patient consciousness to obtain clinical stabilization or perform specific procedures necessary to support the treatment. Optimal sedation has been described as a state in which the patient is sleepy, responsive to the environment, and without excessive movement [1]. To achieve the optimal level of sedation in critically ill patients, doses of sedatives may be individually titrated to the expected effect. This process has been guided by scores on a variety of observational sedation scales [2].

To perform several procedures that require anesthesia or analgesia in pediatric intensive care unit (PICU) scenarios, the identification of the patient’s hemodynamic state has been required. In this context, numerous psychotropic drugs are eligible for this purpose, such as ketamine, which has been claimed as excellent sedation in the intubation procedures in critically ill infants, mainly in the state of hemodynamic instability, which has been referred to as the second-line treatment, according to the Society of Critical Care Medicine Clinical Practice Guidelines (SCCM) [3].

Ketamine use in the PICU context has increased, especially in the pediatric population, due to its minimal cardiovascular effects and bronchodilator effects [4], mediated by its sympathomimetic action and possible modulation of the inflammation cascade [5]. Controversial studies discuss its sympathomimetic action, and recent findings claim that ketamine exerts indirect effects on beta-2 adrenergic receptors [6,7]. Such a mode of action consists of an advantage on cardiorespiratory comorbidities (i.e., bronchospasm) among pediatric patients that require sedation [8,9]. However, administration of higher doses of ketamine presents limited use due to hallucination symptoms in humans and cell death in immature neurons [8,9,10]. Unfortunately, scarce studies have documented the consequences of ketamine sedation procedures in pediatric critical care departments. In this context, our group has claimed such a possibility through a case report published, in which we found that ketamine sedation for 7 consecutive days in a critically ill patient induced long-term behavioral and cognitive consequences, particularly related to language domains, even after hospital discharge and home environmental stimuli [11].

The most important changes in the child’s brain structure and functions occur during the central nervous system development and maturation from birth to adolescence. Particularly during the first 4 years of life, brain structures undergo modifications in morphology, volume, composition, and function [12]. These central nervous system changes are fundamental for the adequate networks of neural connections of cognitive, motor, and sensory functions [12]. In addition, environmental factors such as exposure to psychotropic substances interfere with the brain maturation process, which may provoke central nervous system function impairment that deserves further investigation [13]. There are scarce experimental or clinical studies that support the empirical use of ketamine in PICU. In addition, the control of ketamine activities and its consequences in the short, mid, and long term on critically ill pediatric patients require extensive exploitation. 

This study aimed to conduct a global, bibliometric-type survey to assess relevant metric data on the scientific production about ketamine use in PICU, as well as to provide a global perspective on major clinical designs, authors, countries, etc. In addition, a gap in the literature that deserves further investigation was offered.

## 2. Materials and Methods

To perform this bibliometric analysis, we used the methodology previously described by de Souza Né et al. [14].

### 2.1. Data Source and Collection

**Figure 1:**
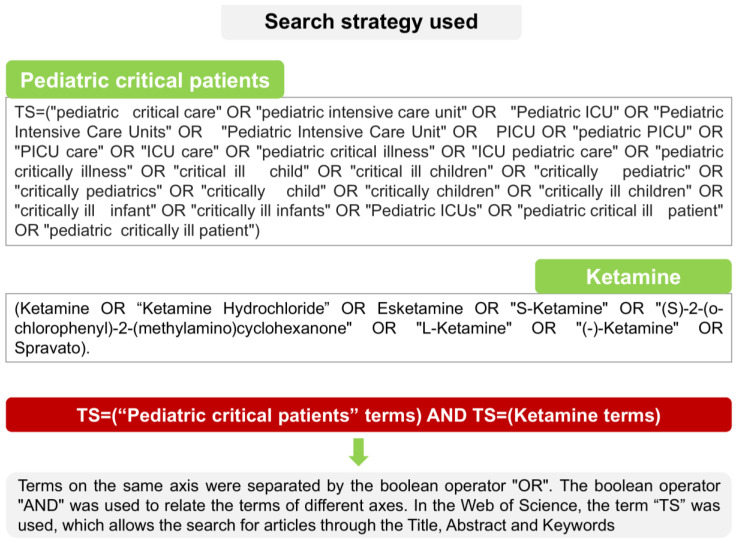
Schematic of search strategy.

A global search was performed on ketamine in the context of pediatric critical care patients in the Web of Science Core Collection (WoS-CC) database. The search strategy applied to retrieve the articles is shown in Figure 1.

### 2.2. Inclusion and Exclusion Criteria

The types of documents selected consisted of original and review articles. No restrictions of language were applied. Finally, the exclusion criteria consisted of conference papers, editorials, letters, papers not available, and publications in which the central theme of the study (ketamine and PICU) was not explored.

### 2.3. Data Selection

To ensure the quality of the selection, two independent researchers searched for articles through the WoS-CC platform. In cases of doubt, a senior researcher was consulted to define the inclusion or exclusion of the study. To optimize the process of extracting articles, two other researchers also participated in the selection phase to collect and compare the number of citations in additional databases (Scopus and Google Scholar). After selecting the articles, a text file generated by the WoS-CC platform was obtained.

### 2.4. Data Analysis

#### 2.4.1. Bibliometric Approach

Bibliometrics is a science that explores the measurement of scientific progress [15]. Three principles underlie a bibliometric study: (1) Bradford’s Law, which relates the prestige of the journal according to the number of citations; (2) Lotka’s Law, which analyzes the scientific productivity of authors; and (3) Zipf’s Law, which evaluates the frequency of keywords [15,16,17]. In this review, we retrieved the following data: articles’ titles, author’s name, number of citations, journal’s name, author’s keyword, countries, and institutions.

Based on data provided by the WoS-CC database, we used the VOSviewer software (version 1.6.16) to obtain interaction networks about co-authorship analysis (considering the amount of publications and citations), occurrence of keywords, and the contribution of institutions. The generated networks must be interpreted as follows: each cluster represents an analysis item (authors, keywords, or institutions); the larger the cluster, the greater the publication/citation of authors, frequency of occurrence of keywords and institutions; the lines between each cluster represent the co-authorship network, connection between keywords, or inter-institutional connection [14]. We also evaluated the relevance of journals, using as a parameter the frequency of publications and visualization of impact factor (considering the JCR 2021) [18]. To assess the worldwide distribution of selected articles, we used the MapChart tool (https://mapchart.net/ accessed on 4 May 2023).

#### 2.4.2. Critical Analysis

**Figure 2:**
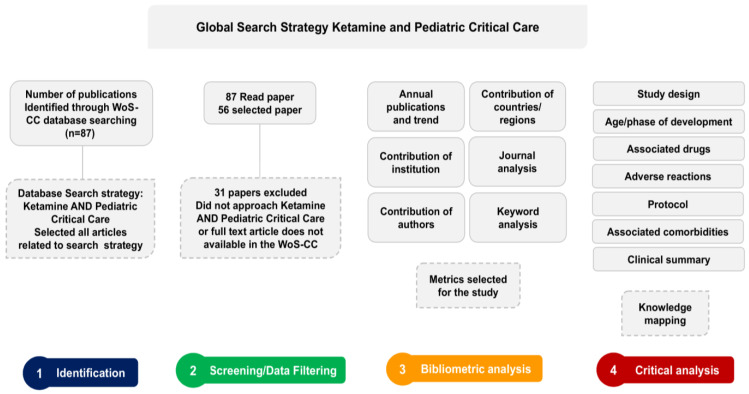
Methodological procedures strategy flowchart.

In the present review, in addition to the conventional metrics of a bibliometric study, we performed a critical clinical findings analysis. Excel software was used to organize the information needed to construct the critical analysis of knowledge. Thus, all selected articles were submitted to a critical evaluation to collect information about the study design, age/phase of development, the regimen of other psychotropic drugs associated with ketamine, adverse reactions, administration protocol, and the clinical summary. Considering that many articles were not precise regarding the classification of the study design, we adopted the classification defined by Nascimento et al. [19]. This analytical evaluation aims to offer a robust survey regarding the use of ketamine in critically ill pediatric patients. Figure 2 summarizes the methodological strategy adopted in this critical analysis.

## 3. Results

### 3.1. Bibliometric Analysis

Through the bibliometric survey performed in the WoS-CC, 87 articles were obtained, of which 56 were selected from the reading of the content, which began by reading the title and abstract (when necessary, the articles were read in full) (Figure 2). A total of 31 articles were excluded for not meeting the established inclusion criteria (Supplementary Materials).

The oldest article was published in 1990 [20] and addressed the use of ketamine in the PICU as a strategy to reduce the use of benzodiazepines. The most recent article, published in 2023, also evaluated the use of ketamine for PICU services and, despite validating the safety and efficacy of the drug, it highlights the importance of studying the long-term effects associated with the use of ketamine in infants [21]. The most cited article consists of a literature review published in 2000 [22], which, at that time, already shed light on the probable negative repercussions of sedoanalgesic procedures during childhood (Table 1).

**Figure 3:**
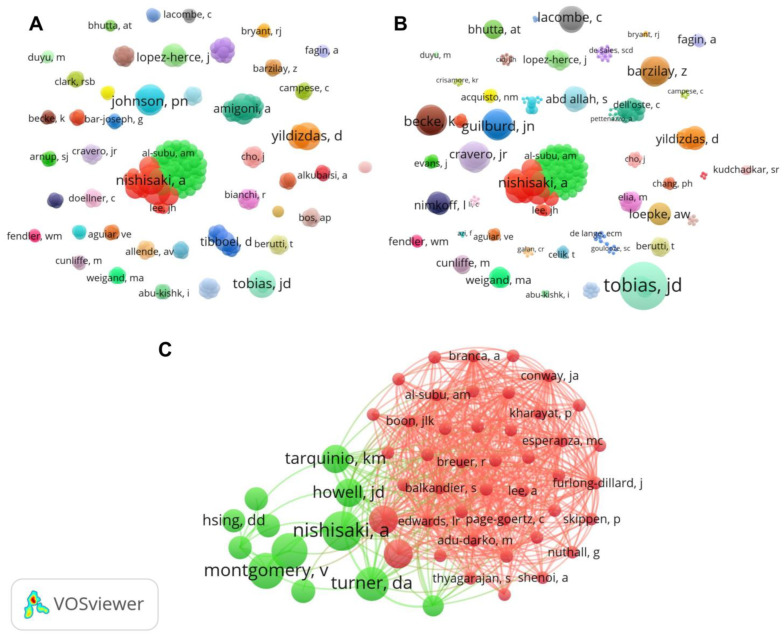
Network visualization of authors with the number of publications (**A**), citations (**B**), and leading network of authors (**C**). There is a direct proportionality of the cluster size and the number of publications or citations.

According to researchers, 287 authors contributed at least 1 article (Figure 3A). The higher numbers of articles were written by Tobias, J.D., Nishisaki, A., Turner, D.A., Yildizdas, D., Johnsom, P.N., and Miller, J.L. (*n* = 3 per author). Regarding the number of citations, Tobias, J.D. (*n* = 313) also represents the author that received the higher number of citations, followed by Becke, K. (*n* = 108), Engelhard, K. (*n* = 108), Sinner, B. (*n* = 108), Bar-Joseph, G. (*n* = 101), Guilburd., J.N. (*n* = 101), Guilburd, N. (*n* = 101), Tamir, A. (*n* = 101), and Nishisaki, A. (*n* = 95) (Figure 3B). The most relevant co-authorship networks were performed by Nishisaki, A. (Figure 3C).

**Figure 4:**
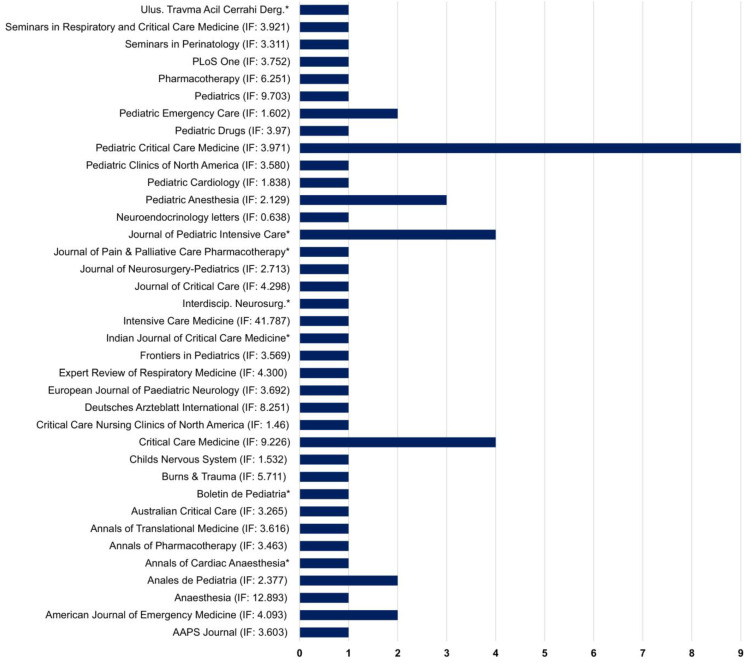
Journals that published the articles focused on clinical studies of ketamine and pediatric critically ill patients. * Journals without impact factor (IF).

According to journals, only 7 journals of a total of 37 periodicals published at least two articles (Figure 4). Among them, the journal Pediatric Critical Care Medicine (*n* = 9; JCR impact factor: 3.971) exhibited the highest number of published articles, followed by the journal Critical Care Medicine (*n* = 4; JCR impact factor: 9.226) and the Journal of Pediatric Intensive Care (*n* = 4). It is important to highlight that both Critical Care Medicine and Pediatric Critical Care Medicine belong to the Society of Intensive Care Medicine, one of the most important and influential entities in the field of pediatric intensive care.

**Figure 5:**
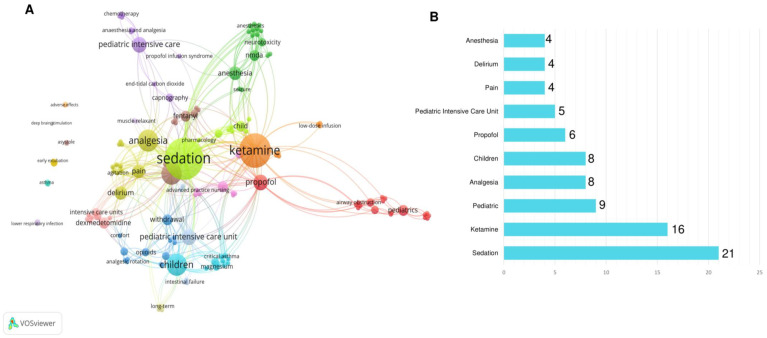
Network visualization of the co-occurrence of the keywords used by the authors of the selected studies using the VOS viewer software. Clusters are highlighted by different colors. The node size represents the frequency of the keyword and the lines reveal the connections between the keywords (**A**). Top 10 most frequent words (**B**).

Keywords represent an important topic of a research article. In this study, a total of 166 keywords were found (authors’ keywords), grouped into 20 clusters (Figure 5A). Figure 5 also exhibits the co-occurrence of keywords through the lines that interconnect the clusters. The top 10 most frequent keywords were sedation (*n* = 21), ketamine (*n* = 16), pediatric (*n* = 9), analgesia (*n* = 8), children (*n* = 8), propofol (*n* = 6), pediatric intensive care unit (*n* = 5), pain (*n* = 4), delirium (*n* = 4), and anesthesia (*n* = 4) (Figure 5B).

**Figure 6:**
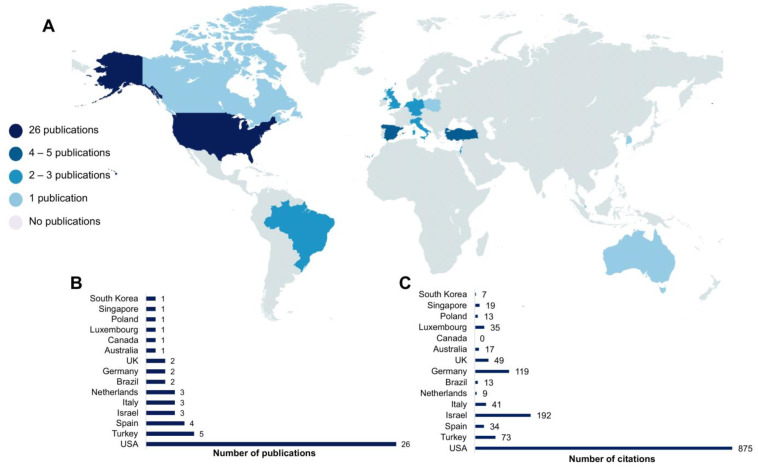
Worldwide distribution of all selected articles (**A**) with the representation of countries from published articles (**B**) and the total number of citations (**C**).

The limited scientific production related to the use of ketamine in pediatric critical patients is distributed in a few countries. Figure 6A,B demonstrate that the United States is the country with the highest number of publications (*n* = 26), followed by Turkey (*n* = 5) and Spain (*n* = 4). Israel, Italy, and the Netherlands reached the mark of three publications. In addition, Brazil, Germany, and the United Kingdom contributed two publications. Regarding citations, the United States (*n* = 867) ranks in first place (Figure 6C). Next, Israel (*n* = 190), even with three publications, occupies the second position (Figure 6C).

**Figure 7:**
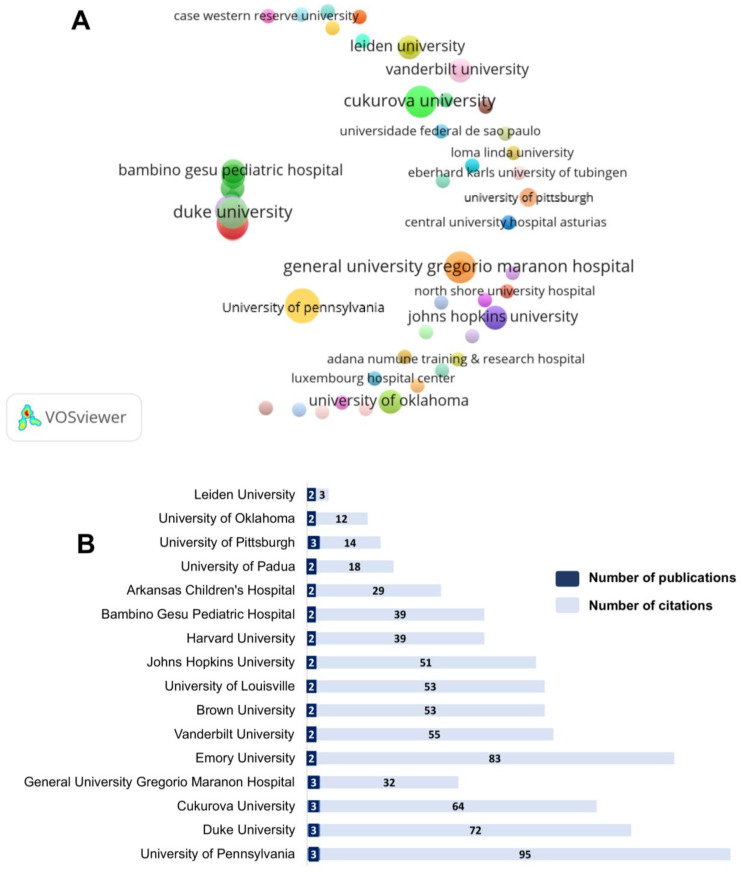
Contribution of institutions (**A**) and top 16 most productive institutions, with the amount of publications and number of citations (**B**). There is a direct proportionality of the cluster size and the number of publications.

A total of 124 distinct institutions, grouped into 43 clusters, were involved in the publication about ketamine in the PICU context (Figure 7A). Only 16 institutions published at least two papers (Figure 7B). The most prolific institutions are concentrated in the United States, totaling 13 institutions (University of Pennsylvania, University of Pittsburgh, Emory University, Vanderbilt University, Brown University, University of Louisville, Johns Hopkins University, Harvard University, Arkansas Children’s Hospital, University of Pittsburgh). These institutions published at least two papers (Figure 7B).

### 3.2. Critical Analysis

**Figure 8:**
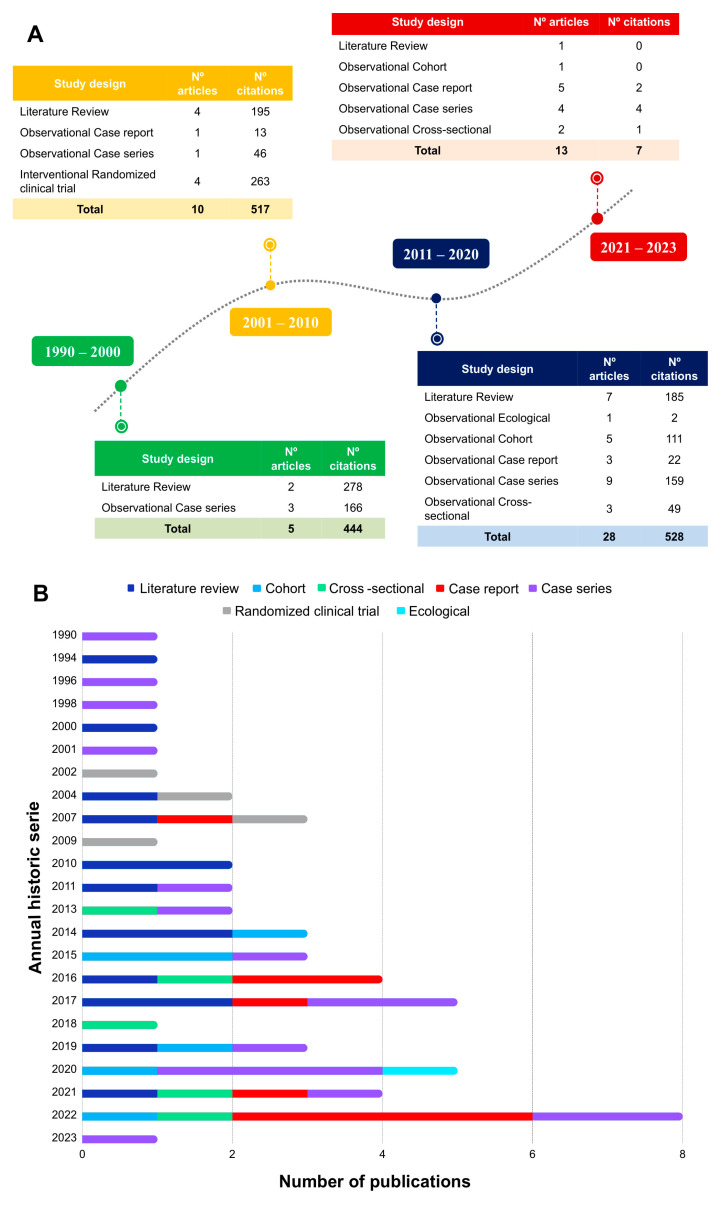
Type of study per decade (**A**) and annual historical series of publications (**B**).

In accordance with the frequency of publication, the period ranging from 2011 to 2020 (*n* = 28) showed the highest number of productions and citations (Figure 8A,B). However, the period ranging from 1990 to 2000 exhibited a lower number of publications (*n* = 5), but a notable number of citations (*n* = 444). Furthermore, case series study (*n* = 17) and literature review (*n* = 14) were the most prevalent studies, with a total of 30 publications. In addition, review articles were the type of study with the highest number of citations (*n* = 658) (Figure 8A,B).

Table 2 shows the clinical findings and other pharmacological issues in the lower number of studies using ketamine on critically ill pediatric patients. Regarding the route of administration, the intravenous route, specifically bolus or continuous infusion, was the principal protocol used. The administration protocols fluctuated between studies, and several of them did not present the dose used. The period of administration also varied between articles, ranging from acute administrations, such as in invasive procedures [33,38], to long-term dosages, such as in cases of patients on mechanical ventilation [11,21,24]. The principal psychotropic drugs associated with ketamine consisted of opioids and benzodiazepines, especially midazolam [11,20,23,24,29,37,40,41,47,63,64,67,68,70,72]. Adverse reactions also varied across the studies, of which dissociative effects, agitation, and cognitive changes compose the main effects described [11,21,22,31,60]. Long-lasting neurobehavioral impairments caused by ketamine have also been reported [11,23]. Regarding comorbidities, respiratory system diseases were the most frequent in these studies [8,11,27,29,40,43,65].

## 4. Discussion

Clinical studies related to the critical pediatric field may support the intensive care services and health professionals in the pharmacological guidelines and procedures in the PICU, such as the sedation and analgesia approach. Thus, this study aims to map the worldwide scientific production presented in the literature about ketamine use in the PICU. The results evidenced the scarcity of publications on this theme, in which the literature review presented a higher number of citations that described the tolerance, physical dependence, and withdrawal of various sedative drugs, including ketamine [22].

### 4.1. Bibliometric Analysis

A bibliometric approach allows mapping the production of scientific knowledge and shedding light on gaps that deserve visibility [73]. Hence, it is necessary to construct a search strategy that is sensitive enough to retrieve all articles related to the study topic. Such a search strategy, in fact, is composed of keywords that represent the subject of the study. Due to the importance of these terms, one of the metrics adopted in this review was based on keyword analysis (Zipf’s Law). We identified that the main keywords present in our study were sedation, ketamine, and pediatrics, which are terms that define the central point of this review. This topic emphasizes the importance of carefully selecting the keywords of a scientific paper.

Regarding Lotka’s Law, by analyzing the scientific knowledge that has been produced about ketamine in the context of intensive pediatrics, a bibliometric approach provides the tendency in the clinical use of ketamine, as well as maps of which research groups have studied the topic. In this regard, our study showed that few researchers (*n* = 287) have studied this relevant issue. We observed that an important co-authorship network, led by Nishisaki, A. [36,51,56], presents a significant contribution to the study theme. In fact, this author is a prolific researcher with several publications in the pediatric field. However, despite this, our study also showed the lack of scientific dialogue among most researchers. In summary, the clinical research on ketamine use in the pediatric intensive care context did not present a consistent science network. This is a dangerous fact, given that preclinical research points to the neurotoxic effect of ketamine on the developing central nervous system [74,75,76,77,78,79,80,81]. Although there are limited translational findings of preclinical studies, these data suggest that the use of ketamine, especially in children, should be carefully controlled.

Bradford’s law assesses the relevance of journals and establishes that a core of journals has greater specificity on a given subject, being more widely cited and relevant [82,83]. In this review, it was possible to identify that the journal with the highest number of publications was Pediatric Critical Care Medicine, followed by Critical Care Medicine, which includes as references in intensive care pediatrics research with higher scientific prestige. Both journals pertain to the Society of Critical Care Medicine, which reinforces the crucial role of professional societies in the development of knowledge among specialists.

North America was the continent with the most articles published around the theme of this report and with independent institutions. This fact might contribute to the difficulty in the standardization of sedation protocols. The theme involving ketamine is still poorly debated since this review showed only 26 published articles on this subject from the USA. In fact, this country features the top research centers with the highest amount of funding for their investigations [84], which justifies the concentration of the most prolific research institutions. Interestingly, Israel stood out as a country with qualified scientific production on the subject, publishing two robust randomized clinical trials. Our survey, however, suggests that this theme is not yet a priority among the main research centers when analyzing scientific production.

### 4.2. Critical Analysis

The critical evaluation of the articles included in the present study demonstrated several cases reports with no description of follow-up after hospitalization in the PICU and the use of a sedative protocol, which may contribute to the identification of the consequences in children’s development. A recent paper published by Sperotto et al. [85] showed that prolonged infusions of ketamine in pediatric critical patients are safe, effective, and reduce the demand for opioids and benzodiazepines. In fact, in clinical practice, there is great concern regarding the use of opioids and benzodiazepines, due to the repercussions generated. Here, we point out that ketamine, despite promoting ideal sedoanalgesia, also needs to be carefully evaluated, especially regarding long-term effects.

The environmental factors (i.e., psychotropic substance exposure) interfere with the brain maturation process, which can provoke central nervous system impairments, as demonstrated previously [13]. In this context, the Society of Critical Care Medicine Clinical Practice Guidelines in 2022 indicated ketamine as a second-choice sedative adjuvant drug in critical care units, mainly in those pediatric patients with hemodynamic instability [3]. Thus, such an anesthetic protocol requires careful and prolonged monitoring of these infants submitted to sedoanalgesia with ketamine, as well as clinical exploitation.

Fundamentally, ketamine consists of a phencyclidine derivative and possesses dissociative properties, with a therapeutic proposal since the 1960s; it pharmacologically blocks the postsynaptic N-methyl-D-aspartate (NMDA) glutamate receptors [86]. This unique mechanism of action alone was enough to induce neurodevelopment brain disorders; however, ketamine exhibits multiple other targets of action, which disturbs normal physiological neurodevelopment, inducing delirium and abstinence syndrome, among other behavioral consequences [11,30].

As mentioned previously, prolonged exposure to ketamine during brain development induces cell death, especially by a mechanism that involves the upregulation of compensation of subunits of the NMDA receptor, triggering intracellular calcium accumulation, increased oxidative stress, and the activation of nuclear factor kappa B (NF-κB) pathway, which promotes more vulnerability for neurons, even after drug withdrawal [87,88]. Furthermore, significant and persistent reductions in cortical and hippocampal volumes occur after psychotropic exposure early in brain development [89]. Changes in the child’s brain structure and functions during its development and maturation, especially in the first 4 years of life, are fundamental for the adequate networks of neural connections of cognitive, motor, and sensory functions [12]. These statements reinforce the need for research focused on the long-term evaluation of the infants submitted to sedoanalgesia with ketamine, as well as other psychotropic substances.

There are inconclusive findings regarding the use of ketamine in the critical evaluation of the selected articles. Some studies emphasize the safety and efficacy of the use of ketamine [20,22,32], highlight its use to reduce the use of opioids [32,46], and consider ketamine as a safe choice to treat status epilepticus [28]. On the other hand, some studies highlight behavioral and cognitive alterations and long-term negative repercussions following ketamine administration [11,59]. Some investigations consider that the association of ketamine plus midazolam reduces the incidence of adverse effects [47]; however, other research associates this association with higher clinical complications [64]. In addition, most studies did not address the use of ketamine as the central theme of the study.

It is important to emphasize that although the bibliometric analysis gathers important elements of metrics, it does not allow the authors to evaluate the methodological quality of the chosen articles, nor the certainty of evidence of these articles. The results presented here do not allow for decision making regarding protocol choices and/or clinical safety. The mapping performed in this study motivates the development of new primary research, as well as secondary studies with other designs and objectives, such as systematic reviews and scoping reviews.

## 5. Conclusions

It is noteworthy that our investigation demonstrated the limited number of randomized clinical and multicentric studies with a representative sample of infants, specifically evaluating the use of ketamine in the context of pediatric intensive care. In this sense, this bibliometric analysis allows us to point out the need for further studies, with more robust methodological designs that provide better scientific evidence on the use of ketamine in critically ill pediatric patients.

## Figures and Tables

**Table 1:** Selected articles employing ketamine in critically ill pediatric patients.

Authors/Year	Article Title	DOI/URL	Journal	Number of Citations		
				WoS-CC	Scopus	Google Scholar
Soblechero et al., 2023 [21]	Prospective observational study on the use of continuous intravenous ketamine and propofol infusion for prolonged sedation in critical care	10.1016/j.anpede.2023.02.014	Anales de Pediatría	0	0	0
Tessari et al., 2022 [23]	Is ketamine infusion effective and safe as an adjuvant of sedation in the PICU? Results from the Ketamine Infusion Sedation Study (KISS)	10.1002/phar.2754	Pharmacotherapy	0	1	2
Duyu et al., 2022 [24]	Emergency application of extracorporeal membrane oxygenation in a pediatric case of sudden airway collapse due to anterior mediastinal mass: A case report and review of literature	10.14744/tjtes.2021.49383	Ulusal travma ve acil cerrahi dergisi	0	0	0
Alkubaisi et al., 2022 [25]	Deep brain stimulation as a rescue for pediatric dystonic storm. Case reports and literature review	10.1016/j.inat.2022.101654	Interdisciplinary Neurosurgery	0	0	0
Crisamore et al., 2022 [26]	Patient-Specific Factors Associated with Dexmedetomidine Dose Requirements in Critically Ill Children	10.1055/s-0042-1753537	Journal of Pediatric Intensive Care	0	*	0
Taher et al., 2022 [27]	Efficacy and Safety of Prolonged Magnesium Sulfate Infusions in Children With Refractory Status Asthmaticus	10.3389/fped.2022.860921	Frontiers in Pediatrics	0	0	1
Howing et al., 2022 [28]	Resolution of status epilepticus after ketamine administration	10.1016/j.ajem.2021.10.052	The American Journal of Emergency Medicine	0	0	0
Machado-Ferraro et al., 2022 [11]	Long-lasting neurocognitive disorders: a case report of previously undescribed adverse effects after ketamine sedation and analgesia in a pediatric patient	10.21037/atm-21-2292	Annals of Translational Medicine	1	*	2
Dervan et al., 2022 [29]	Sleep Architecture in Mechanically Ventilated Pediatric ICU Patients Receiving Goal-Directed, Dexmedetomidine- and Opioid-based Sedation	10.1055/s-0040-1719170	Journal of Pediatric Intensive Care	4	*	4
Moore et al., 2021 [30]	Extended Duration Ketamine Infusions in Critically Ill Children: A Case Report and Review of the Literature	10.1055/s-0040-1713144	Journal of Pediatric Intensive Care	1	*	2
Goulooze et al., 2021 [31]	Towards Evidence-Based Weaning: a Mechanism-Based Pharmacometric Model to Characterize Iatrogenic Withdrawal Syndrome in Critically Ill Children	10.1208/s12248-021-00586-w	AAPS Journal	0	0	0
Li et al., 2021 [32]	Low-Dose Ketamine Infusion as Adjuvant Therapy during an Acute Pain Crisis in Pediatric Patients	10.1080/15360288.2021.1873216	Journal of Pain & Palliative Care Pharmacotherapy	0	0	0
Ekinci et al., 2020 [33]	Sedation and Analgesia Practices in Pediatric Intensive Care Units: A Survey of 27 Centers from Turkey	10.1055/s-0040-1716886	Journal of Pediatric Intensive Care	2	*	3
Aslan et al., 2020 [34]	Effects of Sedation and/or Sedation/Analgesic Drugs Administered during Central Venous Catheterization on the Level of End-tidal Carbon Dioxide Measured by Nasal Cannula in Our PICU	10.5005/jp-journals-10071-23529	Indian Journal of Critical Care Medicine	1	1	0
Sperotto et al., 2020 [35]	Efficacy and Safety of Dexmedetomidine for Prolonged Sedation in the PICU: A Prospective Multicenter Study (PROSDEX) *	10.1097/PCC.0000000000002350	Pediatric Critical Care Medicine	20	20	22
Conway et al., 2020 [36]	Ketamine Use for Tracheal Intubation in Critically Ill Children Is Associated With a Lower Occurrence of Adverse Hemodynamic Events	10.1097/CCM.0000000000004314	Critical Care Medicine	15	13	15
Rubio Granda et al., 2020 [37]	Sedoanalgesia for procedures in Pediatric Intensive Care Unit (PICU). Pharmacology, side effects and quality control	https://pesquisa.bvsalud.org/portal/resource/pt/ibc-201730	Boletin de Pediatria	0	*	0
Iguidbashian et al., 2020 [38]	Enhanced Recovery and Early Extubation after Pediatric Cardiac Surgery Using Single-Dose Intravenous Methadone	10.4103/aca.ACA_113_18	Annals of Cardiac Anaesthesia	9	7	13
Sanavia et al., 2019 [39]	Sedative and Analgesic Drug Rotation Protocol in Critically Ill Children With Prolonged Sedation: Evaluation of Implementation and Efficacy to Reduce Withdrawal Syndrome *	10.1097/PCC.0000000000002071	Pediatric Critical Care Medicine	23	24	39
Park et al., 2019 [40]	Effects of continuous ketamine infusion on hemodynamics and mortality in critically ill children	10.1371/journal.pone.0224035	PLoS One	7	8	17
Walker et al., 2019 [41]	Pain and Sedation Management: 2018 Update for the Rogers’ Textbook of Pediatric Intensive Care	10.1097/PCC.0000000000001765	Pediatric Critical Care Medicine	14	17	35
Groth et al., 2018 [42]	Current practices and safety of medication use during rapid sequence intubation	10.1016/j.jcrc.2018.01.017	Journal of Critical Care	19	23	48
Flint et al., 2017 [43]	Pharmacokinetics of S-ketamine during prolonged sedation at the pediatric intensive care unit	10.1111/pan.13239	Pediatric Anesthesia	5	8	13
Fagin and Palmieri, 2017 [44]	Considerations for pediatric burn sedation and analgesia	10.1186/s41038-017-0094-8	Burns & Trauma	16	19	30
Ketharanathan et al., 2017 [45]	Analgosedation in paediatric severe traumatic brain injury (TBI): practice, pitfalls and possibilities	10.1007/s00381-017-3520-0	Child’s Nervous System	4	6	13
Neunhoeffer et al., 2017 [46]	Ketamine Infusion as a Counter Measure for Opioid Tolerance in Mechanically Ventilated Children: A Pilot Study	10.1007/s40272-017-0218-4	Pediatric Drugs	10	13	23
Pasek et al., 2017 [47]	Case Study of High-Dose Ketamine for Treatment of Complex Regional Pain Syndrome in the Pediatric Intensive Care Unit	10.1016/j.cnc.2017.01.005	Critical Care Nursing Clinics of North America	0	0	0
Chiusolo et al., 2016 [48]	From intravenous to enteral ketogenic diet in PICU: A potential treatment strategy for refractory status epilepticus	10.1016/j.ejpn.2016.08.004	European Journal of Paediatric Neurology	21	32	37
Miescier et al., 2016 [49]	Delayed sequence intubation with ketamine in 2 critically ill children	10.1016/j.ajem.2015.11.053	The American Journal of Emergency Medicine	1	1	4
Golding et al., 2016 [9]	Ketamine Continuous Infusions in Critically Ill Infants and Children	10.1177/1060028015626932	Annals of Pharmacotherapy	12	19	30
Rosenfeld-Yehoshua et al., 2016 [50]	Propofol Use in Israeli PICUs *	10.1097/PCC.0000000000000608	Pediatric Critical Care Medicine	3	5	13
Tarquinio et al., 2015 [51]	Current Medication Practice and Tracheal Intubation Safety Outcomes From a Prospective Multicenter Observational Cohort Study	10.1097/PCC.0000000000000319	Pediatric Critical Care Medicine	39	36	35
Tolunay et al., 2015 [52]	Cerebral salt wasting in pediatric critical care; not just a neurosurgical disorder anymore	https://pubmed.ncbi.nlm.nih.gov/26812288/	Neuroendocrinology Letters	8	*	13
Kamat et al., 2015 [53]	Pediatric Critical Care Physician-Administered Procedural Sedation Using Propofol: A Report From the Pediatric Sedation Research Consortium Database	10.1097/PCC.0000000000000273	Pediatric Critical Care Medicine	69	76	100
Sinner et al., 2014 [54]	General anaesthetics and the developing brain: an overview	10.1111/anae.12637	Anaesthesia	109	131	172
Wong et al., 2014 [55]	A review of the use of adjunctive therapies in severe acute asthma exacerbation in critically ill children	10.1586/17476348.2014.915752	Expert Review of Respiratory Medicine	19	21	39
Nett et al., 2014 [56]	Site-Level Variance for Adverse Tracheal Intubation-Associated Events Across 15 North American PICUs: A Report From the National Emergency Airway Registry for Children	10.1097/PCC.0000000000000120	Pediatric Critical Care Medicine	42	45	33
Larson et al., 2013 [57]	How does the introduction of a pain and sedation management guideline in the paediatric intensive care impact on clinical practice? A comparison of audits pre and post guideline introduction	10.1016/j.aucc.2013.04.001	Australian Critical Care	17	16	28
Smith et al., 2013 [58]	Pediatric Critical Care Perceptions on Analgesia, Sedation, and Delirium	10.1055/s-0033-1342987	Seminars in Respiratory and Critical Care Medicine	19	22	43
Mencía et al., 2011 [59]	Sedative, analgesic and muscle relaxant management in Spanish paediatric intensive care units	10.1016/j.anpedi.2010.12.002	Anales de Pediatría	11	12	21
Murphy et al., 2011 [60]	General Anesthesia for Children With Severe Heart Failure	10.1007/s00246-010-9832-4	Pediatric Cardiology	14	29	20
Neuhauser et al., 2010 [61]	Analgesia and Sedation for Painful Interventions in Children and Adolescents	10.3238/arztebl.2010.0241	Deutsches Arzteblatt International	35	34	57
Loepke, 2010 [62]	Developmental neurotoxicity of sedatives and anesthetics: A concern for neonatal and pediatric critical care medicine?	10.1097/PCC.0b013e3181b80383	Pediatric Critical Care Medicine	72	89	132
Bar-Joseph et al., 2009 [63]	Effectiveness of ketamine in decreasing intracranial pressure in children with intracranial hypertension Clinical article	10.3171/2009.1.PEDS08319	Journal of Neurosurgery-Pediatrics	103	144	268
Bhutta, 2007 [8]	Ketamine: A controversial drug for neonates	10.1053/j.semperi.2007.07.005	Seminars in Perinatology	52	67	113
da Silva et al., 2007 [64]	Procedural sedation for insertion of central venous catheters in children: comparison of midazolam/fentanyl with midazolam/ketamine	10.1111/j.1460-9592.2006.02099.x	Pediatric Anesthesia	12	17	21
Piotrowski et al., 2007 [65]	Hyperkalemia and cardiac arrest following succinylcholine administration in a 16-year-old boy with acute nonlymphoblastic leukemia and sepsis	10.1097/01.PCC.0000257103.96579.B2	Pediatric Critical Care Medicine	13	22	28
Cunliffe et al., 2004 [66]	Managing sedation withdrawal in children who undergo prolonged PICU admission after discharge to the ward	10.1046/j.1460-9592.2003.01219.x	Pediatric Anesthesia	35	45	84
Yildizdas et al., 2004 [67]	The value of capnography during sedation or sedation/analgesia in pediatric minor procedures	10.1097/01.pec.0000117922.65522.26	Pediatric Emergency Care	62	75	103
Vardi et al., 2002 [68]	Is propofol safe for procedural sedation in children? A prospective evaluation of propofol versus ketamine in pediatric critical care	10.1097/00003246-200206000-00010	Critical Care Medicine	86	112	162
Green et al., 2001 [69]	Ketamine sedation for pediatric critical care procedures	10.1097/00006565-200108000-00004	Pediatric Emergency Care	47	73	101
Tobias, 2000 [22]	Tolerance, withdrawal, and physical dependency after long-term sedation and analgesia of children in the pediatric intensive care unit	10.1097/00003246-200006000-00079	Critical Care Medicine	242	308	441
Lowrie et al., 1998 [70]	The pediatric sedation unit: A mechanism for pediatric sedation	10.1542/peds.102.3.e30	Pediatrics	78	102	131
Youssef-Ahmed et al., 1996 [71]	Continuous infusion of ketamine in mechanically ventilated children with refractory bronchospasm.	10.1007/BF02044126	Intensive Care Medicine	51	72	104
Tobias et al., 1994 [72]	Pain management and sedation in the pediatric intensive-care unit	10.1016/s0031-3955(16)38873-3	Pediatric Clinics of North America	36	63	80
Tobias et al., 1990 [20]	Ketamine by continuous infusion for sedation in the pediatric intensive-care unit	10.1097/00003246-199008000-00004	Critical Care Medicine	37	60	83

WoS-CC: Web of Science Core Collection; * Paper not found in the database.

**Table 2:** Critical clinical and pharmacological findings analysis of the selected articles on ketamine and pediatric critical care.

Authors/ Year	Design	Ketamine Protocols	Drugs Associated	Adverse Reactions	Age/ Development Phase	Associated Comorbidities	Clinical Summary
Soblechero et al., 2023 [21]	Case Series	Continuous infusion (IV); 1−2 mg/kg/h; 5 days	Propofol	Hypertension, tachycardia, arrythmia, bronchorrhoea, nystagmus, agitation, and delirium	Average of 6 months old	Unspecified	In this case series study, the authors investigated the association of ketamine and propofol, evaluating the safety and efficacy of this sedoanalgesic association of continuous use in pediatric patients. The authors reported that the observed adverse reactions were short and tolerable, but highlighted the need for robust studies with long-term evaluation to investigate this combination.
Tessari et al., 2022 [23]	Case Series	Continuous infusion (IV); 15–30 ug/kg/min; ≥12 h	Opioids and benzodiazepines	Hypersalivation, systemic hypertension, dystonia/dyskinesia, tachycardia, and agitation	Under 18 years old	Unspecified	In this observational study, the adverse effects associated with the use of ketamine are considered minor and reversible. The authors considered ketamine as an effective and safe drug.
Duyu et al., 2022 [24]	Case Report	Intravenous; Unspecified dose; Single administration	Midazolam	Unspecified	4 years old	Lung cancer	In this case study, cardiorespiratory changes were observed in the patient after the administration of the sedation protocol used (ketamine plus midazolam), emphasizing the risks associated with catastrophic anesthesia.
Alkubaisi et al., 2022 [25]	Case Report	Intravenous; Unspecified dose; 14 days of administration	Dexmedetomidine and clonidine	Unspecified	7 years old	Dystonic storm and cerebral palsy	In this case report, ketamine was used associated with midazolam to attenuate the intense muscle spasms suffered by the patient refractory to sedation with clonidine and clonazepam.
Crisamore et al., 2022 [26]	Cross-sectional study	Intravenous; 0.2 mg/Kg/day; Unspecified period of adm	Dexmedetomidine	Unspecified	Average of 18 months old	Complex chronic condition (CCC)	In this observational study, the authors performed a survey based on the use of Dexmedetomidine in critically ill infants. The collected data showed that the co-administration of other sedatives, such as ketamine, can increase Dexmedetomidine doses. In summary, the authors suggested that a tolerance mechanism would be potentiated by the association of sedatives.
Taher et al., 2022 [27]	Cohort Study	Intravenous; Unspecified dose; Up to 2 days of administration	Magnesium sulfate	Unspecified	Between 8 and 9 years old	Asthma	In this cohort, the authors evaluated asmatical patients with magnesium sulfate and the impacts of this association with other medications, including ketamine, which in turn caused damage but did not compromise the condition of evaluated patients.
Howing et al., 2022 [28]	Case Report	Intravenous; 1 mg/Kg; single dose	Diazepam, fosphenytoin, levetiracetam, lorazepam and midazolam	Unspecified	9 months old	Status epilepticus	In this study, the authors presented a case report of a child with status epilepticus (SE). Ketamine was applied to attenuate SE-evoked seizures. In this case, the authors proposed an interesting clinical use of ketamine and highlighted the need for more randomized clinical trials to prove the efficacy and safety of ketamine in the treatment of SS.
Machado-Ferraro et al., 2022 [11]	Case Report	Intravenous; average of 600 mg/kg; 7 days of administration	Midazolam, fentanyl, and dexmedetomedine	Long-term behavioral and cognitive changes	18 months old	COVID-19	This study evaluated ketamine exposure and its effects in an 18-month-old patient. The patient exhibited behavioral, motor, and cognitive alterations after prolonged use of ketamine.
Dervan et al., 2022 [29]	Case Series	Unspecified protocol	Opioids, benzodiazepines, and dexmedetomidine	Sleep interruption	Average 2.5 years old	Acute respiratory failure	In this study, the authors evaluated the sleep architecture of pediatric critical patients, in which ketamine was associated with increased sleep interruption.
Moore et al., 2021 [30]	Case Report	Intravenous; 5 µg/kg/min; 41 days of adm (case 1); Intravenous; 15–25 µg/kg/min; 33 days of adm (case 2)	Dexmedetomidine, fentanyl, methadone, midazolam and morphine (case 1); Dexmedetomidine, diazepam, hydromorphone, midazolam, and morphine (case 2)	Agitation plus withdrawal syndrome (case 1); Not evaluated (case 2)	2 months old (case 1) and 17 months old (case 2)	Cardiovascular disease (case 1) and tracheoesophageal fistula (case 2)	In this study, the authors presented two case reports on the prolonged use of ketamine in the PICU. It was noticed that, after a prolonged administration of ketamine, children developed ketamine withdrawal, characterized by symptoms of allodynia, hyperalgesia, anxiety, sweating, and drowsiness.
Goulooze et al., 2021 [31]	Literature review	*	*	*	Pediatrics	*	In this study, a secondary analysis originating from an observational study previously published by the authors was performed. Furthermore, a model was proposed to evaluate a recurrent problem in the PICU: the withdrawal syndrome. Then, through the developed model, the authors elucidated that the higher the dose of ketamine, the more days are needed for weaning. In summary, the authors suggested prolonging the weaning period to decrease withdrawal symptoms.
Li et al., 2021 [32]	Case Series	Intravenous; Doses ranging from 0.1 to 0.3 mg/kg; Average of 85.6 h of administration	Opioids	Blood pressure elevation and hallucination	Average 11 years old	Acute pain	In this study, the authors evaluated the application of ketamine as an analgesic adjuvant in pediatric critical patients. Ketamine decreased paintings of pain and reduced the use of opioid drugs.
Ekinci et al., 2020 [33]	Ecological Study	Intravenous; Unspecified dose; Unspecified period of adm	Opioids	Unspecified	Unspecified	Unspecified	This observational study carried out a multicenter survey with the objective of compiling the sedoanalgesic strategies of these centers. The authors observed that ketamine was the first choice for sedonalgesia in short-term procedures. Data on clinical adverse effects were not collected.
Aslan et al., 2020 [34]	Case Series	Intravenous; 1 mg/kg; Unspecified period of time	Unspecified	Hypercarbia and hypoxemia	Average 6.3 years old	Unspecified	In this study, sedoanalgesic drugs were evaluated against a measurement of expired carbon dioxide levels, but the study does not show any clinical results directly associated with ketamine.
Sperotto et al., 2020 [35]	Case Series	Unspecified	Dexmedetomidine	Unspecified	Average 13 years old	Unspecified	In this study, the authors suggested that dexmedetomidine is a safe and effective sedation for PICU patients. In addition, dexmedetomidine was observed to decrease ketamine doses.
Conway et al., 2020 [36]	Cohort Study	Intravenous; Average dose of 1.88 ± 1.12 mg/Kg; Unspecified period of adm	Vagolytic, midazolam, fentanyl, propofol, and neuromuscular blockade	Hypotension, cardiac arrest, and dysrhythmias	Patients < 12 months old and up to 17 years old	Unspecified	In this cohort study, the authors assessed ketamine use on tracheal intubation procedure. The authors associated ketamine with fewer hemodynamic adverse events.
Rubio Granda et al., 2020 [37]	Cross-sectional Study	Intravenous; Unspecified dose; Unspecified period of adm	Midazolam	Hypoxemia	Average 8.3 years old	Unspecified	In this observational study, the authors collected information about sedoanalgesic drugs used in the PICU. According to the survey, ketamine was mainly used in association with midazolam, which did not differ from other sedoanalgesics drugs evaluated.
Iguidbashian et al., 2020 [38]	Case series	Intravenous bolus; 0.5 mg/kg; Intravenous; 0.25 mg/kg/h; Unspecified period of adm	Methadone, lidocaine, acetaminophen, and ropivacaine	Unspecified	Average 7 years old	Unspecified	This study evaluateed methadone as a single-use opioid in extubation protocols. Ketamine was considered a coadjuvant to methadone in postoperative pain relief.
Sanavia et al., 2019 [39]	Case Series	Intravenous; 1 mg/Kg/h up to 2 mg/Kg/h; 3 days of adm	Propofol	Withdrawal syndrome	Average 8 months old	Heart disease, bronchiolitis, traumatic brain injury, sepsis, peritonitis, encephalitis, and leukemia	In this study, the authors developed a rotational sedoanalgesia protocol aiming to reduce the incidence of abstinence syndrome. As a result, in which ketamine was associated with propofol, there was a decrease in the risk of adverse reactions, especially those related to the withdrawal syndrome.
Park et al., 2019 [40]	Cohort Study	Intravenous 8.1 mcg/kg/min; Average of 6 days	Fentanyl, Midazolam, and Dexmedetomidine	Decreased blood pressure; heart and respiratory rates decreased	Average 2.1 years old	Respiratory disease; Cardiac disease; GI/hepatic disease; Other diseases	In this study it was considered that continuous ketamine infusion could be used without hemodynamic instability in PICU patients. There was no statistical difference in mortality rate between the ketamine or non-ketamine groups.
Walker et al., 2019 [41]	Literature review	*	*	*	Pediatrics	*	In this study, articles about pain, sedation, sleep, and delirium in pediatric intensive care were reviewed. Regarding the use of ketamine, the authors report that there is a lack of studies on the use of ketamine exclusively in the PICU, but reinforce the safety of the drug with a low rate of Serious Adverse Events, and as an important agent for sedation; however, the interaction of ketamine with other drugs must be considered with caution.
Groth et al., 2018 [42]	Cross-sectional study	Intravenous bolus; an average of 1.2 mg/kg	Unspecified	hypotension in post-RSI (rapid sequence intubation)	Pediatrics	Unspecified	In this observational study, the authors performed a survey focused on characterizing the sedatives used for immediate intubation. It was found that medication practices during rapid sequence intubation can vary, emphasizing that clinical practice guidelines that provide adequate practices for medication are required. In the evaluated cases, ketamine was used preferentially for induction procedures, without delirium symptoms.
Flint et al., 2017 [43]	Case series	Intravenous: 0.3–3.6 mg/kg/h; Average of 53.5 h	Unspecified	Unspecified	Average of 0.42 years old	Lower respiratory tract infection; encephalopathy; post-surgery; cognitive impairment	In this study, the authors showed that S-ketamine produces unpredictable long-term sedation in children. The interpatient variability in pharmacokinetics complicates the development of adequate dosage regimens. The absence of a control group limits the interpretation of results.
Fagin and Palmieri, 2017 [44]	Literature review	*	*	*	Pediatrics	*	In this literature review, the authors described the challenge of dealing with sedation in critically ill infants. Ketamine has been listed as one of the most commonly used drugs to induce sedation.
Ketharanathan et al., 2017 [45]	Literature review	*	*	*	Pediatrics	*	This literature review presented a compilation of drugs applied in the treatment of traumatic brain injury (TBI). The authors pointed out that ketamine, due to its ability to reduce intracranial hypertension, is a safe alternative for cases of TBI.
Neunhoeffer et al., 2017 [46]	Case Series	Continuous intravenous infusion; Unspecified dose; Average of 3 days	Unspecified	Unspecified	Average of 2.5 years old	Unspecified	In this observational study, the authors aimed to evaluated a protocol which uses ketamine to decrease opioid tolerance. Ketamine, as an adjuvant in sedoanalgesia, was able to decrease the frequency of opioid use and counteract the development of opioid tolerance.
Pasek et al., 2017 [47]	Case report	Intravenous; Unspecified dose; Unspecified period of adm	Ropivacaine, lidocaine, diazepam, and midazolam	Decreased appetite, a mild sensation of bladder fullness, vivid dreams, and drowsiness	Pediatrics	Complex Regional Pain Syndrome (CRPS)	In this study, the authors described that there is no optimal recommendation for ketamine dosing for complex regional pain syndrome (CRPS) therapy. Midazolam was effective to combat the adverse side effects. The midazolam-plus-ketamine association can be very effective; however, it requires toxicity monitoring.
Chiusolo et al., 2016 [48]	Case Report	Intravenous; up to 100 mcg/kg/min	Unspecified	Unspecified	8 years old	Refractory status epilepticus	In this study, the treatment strategy with an intravenous ketogenic diet for refractory status epilepticus was evaluated. The continuous infusion of ketamine did not present efficiency in seizure inhibition. Lacosamide was associated with ketamine but without effect.
Miescier et al., 2016 [49]	Case Report	Intravenous; 1 mg/kg; Single dose (case 1); Intravenous; 2 mg/kg; Single dose (case 2)	Unspecified (case 1); Atropine (case 2)	Cardiovascular adverse reactions (both cases)	11 years old (case 1) and 11 months old (case 2)	Respiratory distress (case 1); Unspecified (case 2)	In this study, the authors reported two cases of critically ill infants who received ketamine. In both cases, the children developed cardiac arrest after ketamine administration. The authors suggested that these catastrophic events were related to the negative inotropic effects of ketamine.
Golding et al., 2016 [9]	Literature review	*	*	*	Pediatrics	*	This literature review highlighted the role of ketamine infusions in critically ill pediatric patients for sedoanalgesia. In this perspective, the authors summarized the main uses of ketamine, which were for sedation, to treat opioid tolerance, and to treat status asthmaticus.
Rosenfeld-Yehoshua et al., 2016 [50]	Cross-sectional Study	Unspecified	Unspecified	Unspecified	Pediatrics	Unspecified	This observational study aimed to survey the use of propofol in PICUs in Israel. However, the data collected by the authors showed that ketamine was considered the safest and most used sedoanalgesia in the PICU.
Tarquinio et al., 2015 [51]	Cohort Stusy	Intravenous; Average 1.9 mg/kg; Unspecified period of adm	Fentanyl, midazolam, and propofol	Hypotension and cardiac arrest	<1 year old; ≥8 years old	Cardiac diseases	In this cohort study, the authors evaluated the medications used in the PICU to perform the tracheal intubation procedure. Among the drugs listed, ketamine was the most used. Furthermore, ketamine was not associated with the development of hypotension.
Tolunay et al., 2015 [52]	Case Series	Intravenous; Unspecified dose and period of adm	Unspecified	Unspecified	14 months old	Cerebral salt wasting syndrome	This case series study described new etiologies for the cerebral salt wasting syndrome (CSWS). The authors claimed that ketamine infusion was classified as the cause of CSWS in one pediatric patient.
Kamat et al., 2015 [53]	Cohort Study	Intravenous; Unspecified dose; Unspecified period of adm	Propofol	Unspecified	Average of 60 months old	Unspecified	This study evaluated a multicenter experience with propofol in critically ill pediatric patients. Among the drugs identified in the psychopharmacotherapy adopted, ketamine was pointed out as responsible for cardiac arrest, without undesirable neurological sequelae. Propofol and ketamine were used concomitantly in 3804 pediatric procedures, however, without specific reports of adverse reactions resulting from this co-administration.
Sinner et al., 2014 [54]	Literature review	*	*	*	Pediatrics	*	In this review, the authors highlighted the possibility that anesthetic drugs produce neurotoxicity in the early stages of development. Thus, the authors carried out a survey of preclinical studies that pointed to ketamine as an inducer of brain damage through a mechanism that involved apoptosis of neurons. Furthermore, it is postulated that more clinical studies, especially randomized clinical trials, should be performed to monitor these possible undesirable neurotoxic effects.
Wong et al., 2014 [55]	Literature review	*	*	*	Pediatrics	*	In this study, the authors reviewed the medications used as adjunctive therapies in acute severe asthma in the PICU. Ketamine has been claimed as an ideal choice for mitigating severe asthma exacerbations.
Nett et al., 2014 [56]	Cohort study	Unspecified	Unspecified	Emesis, hypertension, epistaxis, dental or lip trauma, arrhythmia, pain, agitation, hypotension, and cardiac arrest	Patients < 1 year old and ≥8 years old	Respiratory failure	In this study, the authors evaluated the main adverse reactions associated with tracheal intubation in the variation of site and medications used. The authors claimed that although fentanyl and midazolam were combined in all places, atropine, ketamine, and propofol have been widely used; however, the changes analyzed in the patients would not be associated with this variation in use.
Larson et al., 2013 [57]	Case Series	Continuous intravenous infusion; Average of 3.7 ± 1.8 mcg/kg/min; Unspecified period of adm	Unspecified	Unspecified	<1 year old; 5 years old <12 years old; ≥12 years old	Heart disease, trauma, and general surgical	In this study, the authors analyzed the impacts of applying a guideline on pain and sedation in clinical practice. The authors report that after the implementation of the guideline, there was a reduction in the infusion of ketamine, which was supposedly related to the application of the guide.
Smith et al., 2013 [58]	Literature review	*	*	*	Pediatrics	*	In this study, data are compiled regarding pediatric critical care, considering aspects such as sedation, analgesia, and delirium. Ketamine was described as a safe and effective drug for inducing intubation in critically ill patients. Subhypnotic doses of ketamine administered by continuous infusion decrease the total required opioid and other sedative doses.
Mencía et al., 2011 [59]	Cross-sectional Study	Bolus intravenous and continuous intravenous infusion; Unspecified dose; Unspecified period of adm	Midazolam	Unspecified	Pediatrics	Unspecified	In this observational study, the authors applied a questionnaire to evaluate the drugs used as analgesics, muscle relaxants, and sedatives in PICUs in Spain. The authors observed that ketamine was one of the most used drugs as a sedoanalgesic, usually associated with midazolam and used as an anesthetic, mainly in the intubation of asthmatic patients.
Murphy et al., 2011 [60]	Case Series	Intravenous bolus; 2.4 mg/kg; Intravenous; 1 to 4 mg/kg/h; Unspecified period of adm	Opiates, neuromuscular blocking drugs, volatile anesthetics	Unspecified	Average of 21 months old	Severe heart failure	In this study, the authors suggested that the association of ketamine, opioids, neuromuscular blockers, and volatile anesthetics is relevant for general anesthesia in children with severe heart failure. Ketamine was used in 90% of the studied cases.
Neuhauser et al., 2010 [61]	Literature review	*	*	*	Pediatrics	*	This study addressed sedoanalgesia in painful procedures and emphasizes the importance of guidelines from professional societies of anesthesiology and pediatrics. The study revealed the combination of ketamine and midazolam with a lower rate of complications.
Loepke, 2010 [62]	Literature review	*	*	*	Pediatrics	*	This review compiled articles related to the long-term negative repercussions of sedoanalgesics. The author claims that the clinical current available literature was insufficient. Although the present review was not focused on animal models, it is crucial to report that this review postulated that among the drugs investigated in animal models, ketamine had the highest number of long-term negative effects. These results, although preliminary, shed light on the danger of using sedoanalgesics in critical stages of development.
Bar-Joseph et al., 2009 [63]	Randomized clinical trial	Intravenous; 1–1.5 mg/kg (observation for 10 min)	Midazolam and morphine	Unspecified	Average 7 years old	Unspecified	In this clinical trial, the authors proposed an intriguing research question based on the anecdotal fact that ketamine causes elevated intracranial pressure. The results showed contradictory effects in which ketamine reduced intracranial pressure. The authors also claimed that ketamine is a safe anesthetic agent for patients with traumatic brain injury and intracranial hypertension.
Bhutta, 2007 [8]	Literature review	*	*	*	Pediatrics	*	In this review, there was a survey about the therapeutic properties of ketamine. The authors explained the therapeutic use of ketamine in sedoanalgesia, highlighting important works that validate the effectiveness of ketamine in the intensive pediatric field, also reinforcing its toxic effects, especially related to neurodevelopment.
da Silva et al., 2007 [64]	Randomized clinical trial	Intravenous; 1.40 ± 0.72 mg/kg; 105 min (total sedation time)	Midazolam	Excessive secretion, desaturation, hiccups, transient partial, airway obstruction	Age ranging from 3 to 168 months old	Unspecified	This clinical study aimed to draw a comparative profile between sedoanalgesia induced by midazolam–fentanyl and midazolam–ketamine protocols. The second association, which includes ketamine, exhibited effectiveness in patient stabilization. However, children who received the midazolam–ketamine association presented greater clinical complications. Although these complications were short-lived, these findings shed light on possible negative repercussions associated with the use of ketamine in pediatric patients.
Piotrowski et al., 2007 [65]	Case Report	Intravenous; 50 mg; 15 days of administration	Pancuronium, propofol, and succinylcholine	Respiratory insufficiency and muscle weakness	16 years old	Klebsiella pneumoniae sepsis	In this article, a case of hyperkalemia due to the administration of succinylcholine was observed, and the patient was intubated with a combination of ketamine and other drugs, observing the potassium levels.
Cunliffe et al., 2004 [66]	Literature review	*	*	*	Pediatrics	*	In this study, the authors presented a strategy to decrease the occurrence of abstinence. The authors reported that although ketamine was not related to the withdrawal syndrome, some reports showed that some patients developed a certain tolerance to the drug, requiring an increase in the dose of ketamine to obtain the same effect.
Yildizdas et al., 2004 [67]	Randomized clinical trial	Intravenous; 1 mg/kg; Unspecified period of adm	Midazolam	Respiratory depression	Average of 8.3 and 3.7 years old	Unspecified	This study aimed to analyze carbon dioxide levels with different sedoanalgesics, including ketamine. The results showed that there were no significant differences between the ketamine group and the other anesthetics group. However, the ketamine group was shown to produce a lower incidence of respiratory depression.
Vardi et al., 2002 [68]	Randomized clinical trial	Intravenous; 2 mg/kg; Unspecified period of adm	Midazolam and fentalyl	Apnea, hypotension, hallucinations, airway repositioning	1 month to 28 years old	Unspecified	This study focused on the efficacy and safety of propofol use in the PICU. For this, the authors performed a comparison between the propofol and ketamine groups. Ketamine was less effective than the propofol group.
Green et al., 2001 [69]	Case series	Intravenous; 1.8 mg/kg; Intramuscular; 3.06 mg/kg; Unspecified period of adm	Unspecified	Airway complications, emesis, excessive salivation, and hypoxemia	Average 3.5 years old	Unspecified	This study described the use of ketamine for sedation in children, which claims that ketamine presents security and effectiveness.
Tobias, 2000 [22]	Literature review	*	*	*	Pediatrics	*	This study aimed to observe the tolerance, physical dependence, and withdrawal of various sedoanalgesics. Ketamine, which exhibits antagonistic properties on NMDA receptors, supports the hypothesis of being an important agent in sedation, such as reducing the development of tolerance to opioids.
Lowrie et al., 1998 [70]	Case Series	Intravenous; 1 mg/kg; Unspecified period of adm	Midazolam, propofol	Unspecified	Average 5.6 years old	Unspecified	This retrospective study reported clinical experiences with sedoanalgesic drugs, in which the authors realized that one of the most used drugs in the clinic was ketamine, especially as an analgesic agent, in addition to highlighting its benefits in sedation protocols.
Youssef-Ahmed et al., 1996 [71]	Case Series	Intravenous; 2 mg/kg; Average of 40 h	Albuterol, midazolam	Brief hallucinations, tachycardia, and hypertension	Average 6 years old	Refractory bronchospasm	This retrospective study analyzed children with bronchospasm treated with continuous infusion of ketamine and the authors proposed the hypothesis that this therapy is effective. Continuous ketamine infusion in these patients has been observed to improve gas exchange and dynamic chest compliance.
Tobias et al., 1994 [72]	Literature review	*	*	*	Pediatrics	*	In this review the authors discussed the use of sedoanalgesics and provided an overview of the adverse reactions, use, and protocols.
Tobias et al., 1990 [20]	Case series	Intravenous bolus; 0.5–1.0 mg/kg; continuous infusion (IV); 10–15 mg/kg-min.	Diazepam, midazolam, and fentanyl	Acute epiglottitis and cancer	18 months old to 14 years old	Unspecified	In this case series, the authors aimed to study some sedoanalgesic drugs and report that the use of ketamine in continuous infusion is safe and provides effective analgesia and sedation, without the presence of various irreversible effects.

PICU: Pediatric Intensive Care Unit; ICU: Intensive Care Unit; Adm: Administration; * For review articles, we collected only the clinical summary and standardized the “Age/Development phase” column with the term “pediatrics”.

## Data Availability

All data generated in this review are included in this paper. Further enquiries can be directed to the corresponding author.

## Supplementary Materials

The following supporting information can be downloaded at: https://www.mdpi.com/article/10.3390/jcm12144643/s1, Table S1: List of excluded articles.
