# Supplementary material for: Ketamine Clinical Use on the Pediatric Critically Ill Infant: A Global Bibliometric and Critical Review of Literature
Source: J Clin Med. 2023 Jul 12;12(14):4643. doi: 10.3390/jcm12144643 (PMC10380297; doi:10.3390/jcm12144643)
Supplement: Supplementary file 1 [file jcm-12-04643-s001.zip › jcm-2342052-supplementary.pdf]

Supplementary Information

Ketamine Clinical Use on the Pediatric Critically Ill Infant: A Global Bibliometric and Critical Review of Literature

Mary Lucy Ferraz Maia [1], Lucas Villar Pedrosa Silva Pantoja [1], Brenda Costa Da Conceição [1], KissilaMárvia Machado-Ferraro [1], Jackeline Kerlice Mata Gonçalves [1], Paulo Monteiro Dos Santos-Filho [1], Rafael Rodrigues Lima [2], Enéas Andrade Fontes-Junior [1] and Cristiane Socorro Ferraz Maia [1,*]

.

[1] Laboratory of Pharmacology of Inflammation and Behavior, Faculty of Pharmacy, Institute of Health Science, Federal University of Pará, Belém 66075-900, Pará, Brazil; mary.maia@icb.ufpa.br (M.L.F.M.); kissila.machado@ics.ufpa.br (K.M.M.-F.); jackelinekerlice@gmail.com (J.K.M.G.); efontes@ufpa.br (E.A.F.-J.)

[2] Laboratory of Functional and Structural Biology, Biological Science Institute, Federal University of Pará, Belém 66075-110, Pará, Brazil; rafalima@ufpa.br

* Correspondence: crismaia@ufpa.br; Tel.: +55-9132-01-7201

CONTENTS

Table S1. List of excluded articles.

Article title	Justificative
Exploring a case of incompatibility in a complex regimen containing Plasma-Lyte 148 in the pediatric intensive care	Does not focus on clinical application
Effect of dexmedetomidine and propofol sedation on the prognosis of children with severe respiratory failure: a systematic review and meta-analysis	Does not focus on the use of ketamine
The use of ketamine during intubation of critically ill children with neurologic conditions	Meeting abstract
Multicenter retrospective review of ketamine use in pediatric icu patients (ketamine-picu study)	Meeting abstract
Respiratory adverse events during upper digestive endoscopies in children under ketamine sedation	Article not found
Validation of the Ramsay scale for invasive procedures under deep sedation in pediatrics	Does not focus on the use of ketamine
An Evaluation of Risperidone Dosing for Pediatric Delirium in Children Less Than or Equal to 2 Years of Age	Does not focus on the use of ketamine
Physico-chemical stability of Plasma-Lyte 148 (R) and Plasma-Lyte 148 (R)+5% Glucose with eight common intravenous medications	Does not focus on the use ketamine
Dose effect of ketamine on hemodynamic outcomes among critically ill children with and without shock	Meeting abstract
Beyond Opioids for Pain Management in Adult Critically Ill Patients	Not related to PICU
A Comparison of Safety and Efficacy of Dexmedetomidine and Propofol in Children with Autism and Autism Spectrum Disorders Undergoing Magnetic Resonance Imaging	Does not focus on the use of ketamine
Role of regional analgesic techniques in ICU	Does not focus on the use ketamine
Treatment, outcomes and costs of asthma exacerbations in Chilean children: a prospective multicenter observational study	Does not focus on the use of ketamine
Role of sedation and analgesia in ICU	Not related to PICU
Dexmedetomidine for Sedation During Noninvasive Ventilation in Pediatric Patients	Does not focus on the use of ketamine

Article title	Justificative
Sedating Children on Extracorporeal Membrane Oxygenation: Achieving More With Less	Editorial material
Sedation and Analgesia for Critically Ill Pediatric Burn Patients: The Current State of Practice	Article not found
Observations on the Effects of Inhaled Isoflurane in Long-term Sedation of Critically Ill Children Using a Modified AnaConDa (c)-System	Article not found
Ketamine for continuous sedation in patients admitted to the pediatric intensive care unit	Meeting abstract
The effects of age, isoflurane and sevoflurane on atracurium in lambs	Not related to PICU
Pediatric fiberoptic bronchoscopy as adjunctive therapy in acute asthma with respiratory failure	Does not focus on the use of ketamine
Pediatric Resuscitation and Cardiac Arrest	Does not focus on the use of ketamine
Ethics roundtable: 'Open-ended ICU care: Can we afford it?'	Not related to PICU
Evaluation and management of moderate to severe pediatric head trauma	Does not focus on the use of ketamine
Magnesium sulfate administered via continuous intravenous infusion in pediatric patients with refractory wheezing	Does not focus on the use of ketamine
Prospective evaluation of propofol anesthesia in the pediatric intensive care unit for elective oncology procedures in ambulatory and hospitalized children	Does not focus on the use of ketamine
Propofol anesthesia for invasive procedures in ambulatory and hospitalized children: Experience in the pediatric intensive care unit	Does not focus on the use of ketamine
The effects of intravenous anesthetics on intracranial pressure and cerebral perfusion pressure in two feline models of brain edema	Does not focus on the use of ketamine
Treatment of severely burned children in a pediatric intensive-care unit	Article not found
Pediatric intensive-care sedation - survey of fellowship training-programs	Article not found
Sedation and analgesia in the pediatric intensive care unit	Article not found
